# From bugs to beta cells

**DOI:** 10.7554/eLife.23065

**Published:** 2016-12-13

**Authors:** Yuxi Zhang, Daniel Hesselson

**Affiliations:** 1 Diabetes and Metabolism Division, Garvan Institute of Medical Research, Sydney, Australia; 1 Diabetes and Metabolism Division, Garvan Institute of Medical Research, Sydney, Australiad.hesselson@garvan.org.au

**Keywords:** microbiota, beta cells, development, Zebrafish

## Abstract

Certain microbes in the intestine secrete protein that stimulates the proliferation of beta cells in the pancreas during development.

**Related research article** Hill JH, Franzosa EA, Huttenhower C, Guillemin K. 2016. A conserved bacterial protein induces pancreatic beta cell expansion during zebrafish development. *eLife* **5**:e20145. doi: 10.7554/eLife.20145

After birth, we are colonized by microbes that quickly outnumber all of the other cells in the human body. There is intense interest in how these microbes, which are collectively known as the microbiota, can promote health or contribute to disease, but comparatively little attention has been paid to their role in development. The importance of the microbiota in the development of the gut was first recognized more than 40 years ago, once it became possible to raise gnotobiotic animals (animals for which only certain known microbes are present) under germ-free conditions (Thompson and Trexler, 1971). Could the development of organs that are not in direct contact with the microbiota also be under its control?

Until recently, our understanding of the microbiota of vertebrates was limited to microbes that could be cultured in the lab. However, advances in microbial taxonomy and DNA sequencing technology have revealed enormous, and previously underappreciated, diversity in the composition and function of the microbiota (Shokralla et al., 2012). Emerging data suggests that the presence or absence of rare microbes could have profound physiological consequences: some strains can reprogram the metabolism of the entire microbial community (McNulty et al., 2011), whereas others produce factors that act directly on the host (Semova et al., 2012).

Zebrafish develop outside of the mother, which makes them an ideal vertebrate system in which to manipulate the composition of the microbiota in order to study its role in development. In addition, the optical transparency of zebrafish and the availability of many tissue-specific transgenic markers provide a window into organ development at cellular resolution. Early studies of germ-free zebrafish highlighted that the microbiota had a conserved role in the growth and maturation of the intestine (Bates et al., 2006; Rawls et al., 2004). Now, in eLife, Karen Guillemin of the University of Oregon and colleagues – Jennifer Hampton Hill (Oregon), Eric Franzosa and Curtis Huttenhower, both of Harvard and the Broad Institute – have used these tools to reveal that microbiota-host interactions could play a role in the development of beta (β) cells in the pancreas (Hill et al., 2016).

Small clusters of hormone-producing cells within the pancreas keep blood sugar levels within a narrow range. The β cells, which produce insulin, are of particular importance because they are dysfunctional in patients with diabetes (or have been destroyed). Hill, Franzosa, Huttenhower and Guillemin (who is also at the Canadian Institute for Advanced Research) noticed that a rapid expansion in the number of pancreatic β cells coincided with the newly hatched zebrafish larvae being colonized by microbes. Using the germ-free system, they discovered that the microbiota is required for this expansion, which is driven by proliferation of existing β cells as well as the differentiation of new β cells (Hesselson et al., 2009).

Hill et al. then took a collection of bacterial strains that they had previously isolated from the zebrafish gut (Stephens et al., 2015), and determined which of these strains promotes β cell expansion in germ-free zebrafish. Only a subset of the strains, including several *Aeromonas* isolates, restored normal β cell numbers. This suggested that a strain-specific factor was involved. The *Aeromonas* activity was tracked to a mixture of proteins that were secreted by the strain in culture, setting off a hunt for the factor (or factors) responsible.

Using genomic and proteomic filters Hill et al. identified a single candidate protein, which they named β cell expansion factor A (BefA). When the *BefA* gene was deleted from the *Aeromonas* genome, the mutant strain still colonized germ-free zebrafish but it did not restore β cell numbers. In contrast, the addition of purified BefA protein to germ-free zebrafish fully rescued β cell development. These elegant experiments showed that BefA is necessary and sufficient to promote β cell expansion, and that it exerts its effect directly on the host. Additional experiments showed that BefA specifically promotes the proliferation of existing β cells and/or progenitor cells that are already committed to the β cell fate.

This discovery in zebrafish could have implications for human health. Homologs of *BefA* exist in the genomes of bacteria that colonize the human gut. Intriguingly, BefA proteins from the human microbiota also stimulated β cell expansion in zebrafish, suggesting that they share an evolutionarily conserved target. Unfortunately, the sequence of the protein does not provide many clues to its biological function.

The intestines and β cells communicate extensively with each other (via hormonal and neuronal signals) to help match food intake with insulin output, and early β cell expansion could be regulated by similar inter-organ signals. Whether BefA acts directly on β cells/progenitors (Figure 1A) or indirectly by rescuing intestinal development or function in germ-free animals (Figure 1B) remains unknown. However, the gnotobiotic zebrafish system is poised to deliver fundamental insights into BefA targets and function.

**Figure 1.**
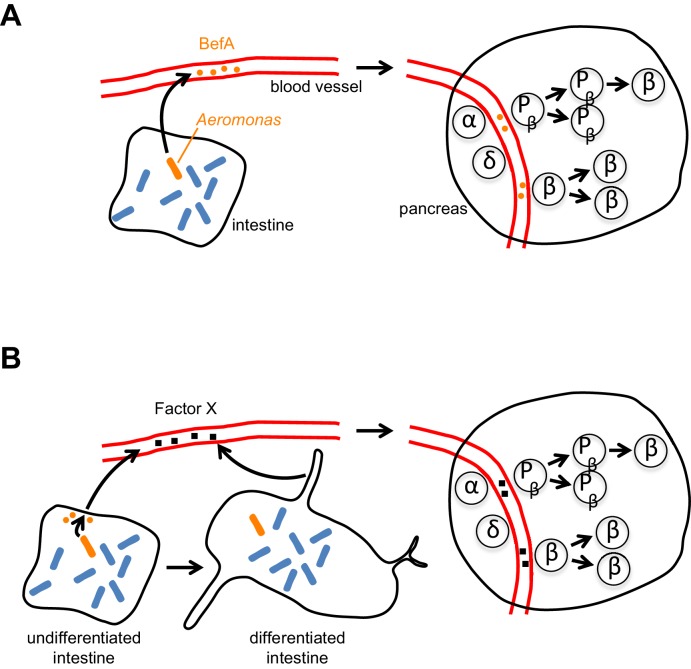
How does BefA stimulate the proliferation of β cells in the pancreas? (**A**) In the direct model certain microbes (such as some strains of *Aeromonas*, a rod-shaped bacterium shown here in orange) in the lumen of the intestine secrete BefA (orange circles), which then circulates in the blood and stimulates the proliferation of β cells and/or β cell progenitors (P_β_) in the pancreas. (**B**) In the indirect model BefA promotes the differentiation of the intestine or stimulates the cells lining the intestine to release an undefined ‘Factor X’ (black squares), which then circulates in the blood and promotes β cell expansion in the pancreas. The α- and δ-cells in the pancreas are not affected by BefA.

By two years of age, the proliferation of β cells in humans slows markedly (Gregg et al., 2012), which suggests that the proliferation of perinatal β cells may be important for establishing a reserve of β cells to promote lifelong metabolic health (Berger et al., 2015). Susceptible individuals with a suboptimal number of β cells may struggle to meet the increased demand for insulin associated with pregnancy or obesity. Determining whether BefA can act at later stages of development to allow β cell numbers to 'catch up' could lead to novel therapeutic approaches for the prevention of diabetes.
